# Acquisition of L2 English spatial deixes by Arabic-speaking children

**DOI:** 10.3389/fpsyg.2022.997110

**Published:** 2022-11-03

**Authors:** Hissah Nasser Alothman, Haroon N. Alsager

**Affiliations:** Department of English, College of Science and Humanities, Prince Sattam Bin Abdulaziz University, Al-Kharj, Saudi Arabia

**Keywords:** spatial deixis, English, second language acquisition, children, Arabic

## Abstract

Deictic words are considered the earliest words which children acquire at the stage of two-word-utterance. However, mastering them like adults may take more time. This paper investigates how L2 children comprehend and produce English spatial deixis ‘*here*’, ‘*there*’, ‘*this*’, and ‘*that*’ by observing and documenting their responses and reactions in hide-and-seek game. It also aims to find out the children’s obstacles in acquiring these words, such as proximity bias and egocentrism. The subjects are Arabic children of ages four, five, and six who acquire English as a second language in international schools in Riyadh, Saudi Arabia. They performed two types of tasks: comprehension task and production task. Both tasks contained two trials: same perspective and the different perspective. Based on the results, children did better in comprehending the spatial deixis than in producing them. Moreover, the results showed that there was no proximity bias happened with children in this study. In addition, the results of the two trials in both tasks illustrated that changing the deictic center improves with age. Although the study provides some significant results, there should be an increase in the number of the samples in order to make the results generalized.

## Introduction

One of the main things which makes participants communicate effectively is to share the knowledge of what they have been talking about. According to Zhao (2007), deictic words are considered the most useful tool which reflect the relationship between language and context. They are used to refer to a location in which objects are in a specific context (Cutting, 2005). Thus, in all languages, the utterance is linked with its context. In the early stages of development, children start communicating and interacting with their caretakers about locations (*here\there*) and objects (*this\that*) around them. The reason of this study’s concentration on deictic words, especially spatial deixis, is that they are challenging our ability to learn\acquire a language.

There are many conducted studies about the first language acquisition of spatial deixis in many different languages. The acquisition of spatial deixis (demonstratives) has become a new subject again in some recent works (for a review see Zhao, 2007; Krasnoshchekova, 2012; Muşlu, 2015; Gonzalez Pena, 2020). Based on our readings, there is a small number of studies which have been conducted about the acquisition of spatial deixis as a second language. Although most of the studies were conducted on children’s acquisition of deictic words as a first language, this study tries to focus on the L2 children’s comprehension and production of English spatial deixis. The present study helps us to understand the development of children’s acquisition of spatial deixis. The deictic words which we will focus on in the current study are *here\there* and *this\that.*

This paper poses the following questions: Is there any age effect on children’s comprehension and production of acquiring English demonstratives and locative adverbs as a second language? Do children take the speaker as a point of reference? Do children first acquire the proximal deixes ‘*this\here*’ or the distal ones ‘*that\there*’? Which deictic pair ‘*this\that*’ or ‘*here\there*’ do children acquire first? In order to answer the previous questions, the overall structure of the study is divided into six parts. The first part provided a general introduction of the study topic followed by the research significant and the research objectives. The second part begins with an overview of the main theories of first and second language acquisition, and a general introduction about the differences between Arabic and English demonstratives. Then, the same part reviews some previous studies about children’s acquisition of spatial deixis in different languages, and it mentions some of their main issues. The third part presents the research questions which led to the conduct of this study. The fourth part discusses the method used in this study. The study’s findings are presented in the fifth part. Finally, the last part gives a conclusion with the study’s limitations.

### Research significance

The present study will be valuable to those who teach and acquire English in Arab countries in addition to those who will conduct studies about the acquisition of English spatial deixis. It is also important for parents whose children acquire English as a second language as well. The current research provides information about how children acquire the spatial deixis from age four till six. In the research field, researchers tend to figure out the children’s obstacles in acquiring those spatial deixes. This paper proves that children have some characteristics which may affect their acquisition of languages, especially deixis words.

### Research objectives

The major objectives of the present study are as follows:

1. To find out the children’s obstacles in acquiring English spatial deixes.
2. To find out the developmental stages in acquiring the spatial deixes.

## Literature review

### Demonstratives structure in Arabic and English

At the early stages, infants start interacting with their parents and caregivers about objects and locations in space. As an outcome, they are engaging in deictic communication (Gonzalez Pena, 2020). According to the frequency counts in lexical databases, such as Celex, Lexique and Subtlex, demonstratives are amongst the most highly used lexical items in many languages (Peeters et al., 2021). The number of demonstratives in each language is a remarkable cross-linguistic diversity. In English, demonstratives are classified as ‘proximal’ (*this* and *here*) and ‘distal’ (*that* and *there*) forms. Some languages divided demonstratives into three types, such as Spanish and Japanese. Quileute, Somali, Malagasy and Navajo are languages which divided demonstratives in more than three types (Diessel, 1999, 2013; cited in Peeters et al., 2021). In all languages, demonstratives play a crucial role in any discourse. For example, speakers use demonstratives to refer to either the objects in the context (exophoric use) or things which represented textually (endophoric use or cataphoric use; Zaki, 2007). Because all the study’s participants are Arabs who acquire English as a second language, it is important to mention the main differences between the structure of demonstratives in Arabic and English. The following paragraphs will illustrate types of demonstratives in English and standard Arabic.

In English, ‘*this*’, ‘*that*’, ‘*these*’ and ‘*those*’ are demonstratives, whereas ‘*there*’ and ‘*here*’ are the locative adverbs. The demonstratives ‘*this*’ and ‘*that*’ can be used as pronouns (e.g., ‘*what is that?*’), or determiners (e.g., ‘*that book on the right*’; Rabadi, 2016; Gonzalez Pena, 2020). Demonstrative pronouns and demonstrative adjectives are similar, but they are different in sentence/phrase structure. Table 1 shows that the former is used as a pronoun with a demonstrative meaning, while the latter is used as a determiner which followed by a noun (Rabadi, 2016). In other words, the main difference is that the demonstrative pronoun takes the place of the noun phrase, while the demonstrative adjective (often called determiner) comes before a noun. The demonstrative pronouns\adjectives can be used to indicate objects which are far or near from the speaker (*this, that, these, those*; Musa, 2017).

**Table 1:** The difference between demonstrative pronouns and demonstrative adjectives (Rabadi, 2016).

Demonstrative Pronouns	Demonstratives followed by N
This is a red dress.	This dress is red.
That is a tamed lion.	That lion is tamed.
These are intelligent boys.	Those boys are intelligent.
Those are beautiful roses.	Those roses are beautiful.

Demonstratives in Arabic are more complicated than in English (Gonzalez Pena, 2020). In Standard Arabic, the demonstratives have been described as ‘Names of Reference’ (Musa, 2017). The spatial deixes are demonstratives which are used near the speaker: اولاء، الاء، هنا، هناك، هنالك,, and further away from the speaker: اولئك، اولائك، الائك. For the singular and dual forms, see Tables 2, 3 (Al Aubali, 2015). According to Rabadi (2016), demonstratives in Arabic are divided into two groups which depend on the referent. The first part focuses on the referent recognition in terms of singularity, duality, plurality, muscularity and femininity. The second part focuses on how the referent is proximal, medial and distal. For instance, the prefix ‘hā’ is used to indicate proximity, the suffix ‘ka’ is used to indicate medial distance, and the suffix ‘li’ is used to indicate distal distance. The demonstrative pronouns in Arabic occur before a noun which they refer to; for example, احب ذلك الطالب ‘I like that student’. On the other hand, the demonstrative modifiers\adjectives can occur before (prenominal position) or after (postnominal position) a noun, such as ‘that man’ ذلك الرجل and ‘those girls’ البنات أولئك.

**Table 2:** Arabic demonstratives used near the speaker.

	Masculine	Feminine
Singular	هذا.	هذه.
Dual	هذان، هذين.	هاتين، هاتان.

**Table 3:** Arabic demonstratives used away from the speaker.

	Masculine	Feminine
Singular	.ذاك، ذالك	تلك، تاك.
Dual	ذانك، ذينك.	تينك، تانك.

To sum up, the main difference between Arabic and English demonstratives is that English has only two-dimensional deictic points (distal and proximal), while Arabic has three deictic points (distal, medial and proximal; Rabadi, 2016). Moreover, the demonstrative position is also different in both languages. In English, when a demonstrative is used as a determiner, it precedes the head. On the other hand, it precedes or follows the head in Arabic (Al Aubali, 2015; Alsager, 2017, 2020; Alsager and Mahzari, 2021). For example:

1. I have read this book.

قرأت هذا الكتاب / قرأت الكتاب هذا

1.a qara’atu hatha alketab \ qara’atu alketab hatha.

1.b. I have read this book \ I have read book this.

The main features which are shared by the Arabic and English languages are the proximal and distal features. The proximal form is speaker-oriented while the distal form is hearer-oriented. Both of the languages share the same grammatical features of demonstrative pronouns, but they are different in one way. In Arabic, the demonstratives (proximal and distal forms) are marked in number (singular, dual, plural), gender (masculine, feminine) and case (nominal, genitive, accusative; Zaki, 2007). Zaki (2007) classified the structure of demonstrative phrase in Arabic and English into three categories based on two samples of corpora (see Table 4). The first category contains a demonstrative and a head noun without a modifier. The second category contains a demonstrative phrase with an adjectival modifier before (in English) or after (in Arabic) the head noun. The third category is having the most complex structures where a structure is modified with propositional phrases or relative pronouns with or without adjectives.

**Table 4:** Classification of syntactic structure in Arabic and English (Zaki, 2007).

Syntactic structure	English %	Arabic %
Head Noun only	74	73
Adjective modifying	14	18
Complex modifying	12	9

Although the Arabic and English languages belong to different families, their uses of demonstratives are somehow similar to each other in both semantic and syntactic features. The study of Rabadi (2016) showed that the demonstratives in Arabic and English share some linguistic similarities than differences. In other words, demonstratives in both languages are similar, except for the dual form in the Arabic language. Furthermore, the demonstratives alone in both languages are vague unless they are used in a context.

### Previous studies

Deictic words are considered aspects of language which require sorts of contextualization, such as time, place, and person (Fillmore, 1975). One type of deictic words is spatial deixis. Murphy (1986) defined it as “words that are dependent on the spatial position of the speaker and/or hearer and convey information about location” (p: 119). Spatial deixis contains demonstratives and locative adverbs. Demonstratives have a long history, and they can be found in all languages. They can be deictic or non-deictic. All deictic words are exophoric which are used to refer to objects and locations in the context. On the contrary, the non-deictic words are used to refer to things in the text. Anaphora is the most well-known non-deictic. Demonstrative words in English are the singular ‘*this*’ and ‘*that*’, the plural ‘*these*’ and ‘*those*’, and the locative adverbs ‘*here*’ and ‘*there*’. The main difference between the demonstrative words and the locative words is that the former is used to refer to objects, whereas the latter is used to identify specific places (Clark and Sengul, 1977; Gonzalez Pena, 2020).

The acquisition of deictic words, especially the spatial deixes, requires mastering some main factors. The first factor is about determining who is speaking and who is being addressed. The second factor is to be aware of the speaker and the addressee’s place (Fillmore, 1975). For example, the phrase ‘that book’ for the speaker might mean ‘this book’ for the hearer (De Villiers and De Villiers, 1974). Moreover, Clark and Sengul (1977) added two main factors about spatial deixes. The first factor emphasizes to understand that the speaker is the point of reference; for example, ‘*here*’ refers to a place near the speaker while ‘*there*’ refers to a place farther away. The second factor is that each pair of deictic words contrasts in the distance dimensions; for example, ‘*here*’ and ‘*this*’ are proximal while ‘*there*’ and ‘*that*’ are non-proximal (Clark and Sengul, 1977). According to Zhao (2007), the acquisition of demonstratives is considered a gradual process. All children around the world do not acquire them all at once.

There are many conducted studies about the acquisition of deictic words whether in the first or second language. The acquisition of deixis has been a subject of some recent studies (Zhao, 2007; Krasnoshchekova, 2012; Muşlu, 2015; Gonzalez Pena, 2020). There are several issues which have been discussed regarding the acquisition of deictic words. First, it has been argued that deictic words are among those words that are acquired first. Second, some researchers discussed the main reasons of children’s errors in their studies which lay back to their bias toward choosing objects near them. Third, some researchers conducted studies to see whether cues and gestures affect the children’s performance about deictic words. Fourth, there was also a point which has been discussed about the children’s recognition of deictic contrasts. Fifth, some studies tested children to see the most difficult deictic pair to acquire. Each issue will be discussed in depth in the following paragraphs.

There was a claim which stated that demonstrative words are among the earliest words which children acquire. Caselli et al. (1995) described in their study the language acquisition of English and Italian through parental report with the MacArthur-Bates Communicative Development Inventory (CDI) on more than 800 children. The results showed that the demonstratives did not appear with the first 50 words which children acquired in both languages. Similarly, González-Peña et al. (2020) conducted a study and re-evaluated this claim. The results of the spontaneous speech transcripts and parental report data showed that demonstratives are not the most frequent of early verbal deixis. In other words, they did not appear before the 50th word which children acquired. They are more frequent at the stage of two-word-utterance. Many researchers also faced the problem of children’s proximity bias in their studies. In the experiment of Clark and Sengul (1977), they put two toys within reach of the children in order to avoid bias. They were 30 and 40 away from the children. In addition, there is an egocentrism hypothesis which stated that children who are under the age of six have some difficulties in shifting the deictic center when they and the experimenter have different perspectives. According to many results, the children’s performance in the different perspectives was worse than in the same perspective. They never see things from anybody else’s point of view except their own (Zhao, 2007; Muşlu, 2015). According to Zhao (2007), all children think in an egocentric way. Thus, egocentrism is probably a universal phenomenon which affects the acquisition of a language in children.

There are some devices in every language, such as linguistic deictic terms and extralinguistic devices, which are used to convey deictic aspects of communication. Linguistic deictic terms are like the pronouns of the participants in the discourse, their locations, and the time when the utterance is occurred. On the other hand, extralinguistic devices are like the gestures and the facial expressions which are used by the participants (Tfouni and Klatzky, 1983). According to many studies of spontaneous speech, it is natural that children at a very early-stage point to things by using nonlinguistic cues, such as gestures or eye-gaze. Later, they use deictic words along with their gestures (Clark and Sengul, 1977; Zhao, 2007). There are many different researchers who have discussed the effect of gestures and cues on children’s performance. The two following paragraphs will discuss some of them.

Tfouni and Klatzky (1983) conducted a study which aimed to test the children’s comprehension of the spatial deixes in two different conditions: hearer-addressee and hearer-spectator. The population consisted of 18 children who were divided into two groups: the pragmatic group and the semantic group. The procedure which they used is similar to Clark and Sengul (1977). With the pragmatic group, the experimenter was pointing by using gestures to disambiguate the meaning of the spatial deixis. On the other hand, there were no gestures used with the semantic group. According to the findings of the study, there is a distinction between the children’s comprehension of the linguistic knowledge and the pragmatic knowledge. The pragmatic knowledge of place and space is acquired first, followed by linguistic knowledge. The use of pointing gestures (pragmatic) with the deictic words (semantic) facilitates the comprehension of the utterance in which the words appeared. In addition, children in their study found that when the deictic words were used without gestures, they were difficult to comprehend. They concluded the paper by mentioning the importance of pointing in children’s comprehension because the lexical words which are used to indicate the deictic meaning have only a partial meaning.

The use of cues has also an impact on the children’s ability to understand the demonstratives. Muşlu (2015) conducted a study which aims to provide information about the development of children’s comprehension of demonstrative pronouns in their first language (Turkish). Moreover, the researcher tried to find out whether there is an age effect on the children’s comprehension or not. She also used toys with 12 children ages 3–5. In her study, the children should make a choice between two toys based on the experimenter’s testing sentences. When the testing sentence was with non-linguistic cue, she looked at the child directly without any cues. On the other hand, when the testing sentence was with a linguistic cue, she said the sentence by giving an eye gaze to the objects. The findings of the study revealed that the use of physical cues had an impact on children’s ability to perform correctly. However, the results are varied without any physical cues.

Spatial deixes, such as demonstratives and locative adverbs, show pair contrasts (Fillmore, 1975). Children at the early ages do not recognize the deixis contrasts. Clark and Sengul (1977) conducted a study in which the first experiment aimed to see how children interpret and deal with deictic contrasts. The experimenter set a game where a child decides which toy should he\she move. According to the results of their experiment, children went through three stages in acquiring deictic contrasts. In the first stage, children acquired deixis without any contrast. At this stage, they did not know that the point of reference is the speaker, and the deictic terms contrast on a proximal/non-proximal dimension. Both of the speaker principle and the distal principle were not mastered by the children. In the second stage, they mastered them with partial contrast. In the last stage, they acquired deixis with full contrast equivalent. According to their findings, children acquired the locative pair ‘*here*’ and ‘*there*’ before they acquired the demonstratives ‘*this*’ and ‘*that*’. In short, children improve as they grow older.

In addition, many researches were conducted to see whether the proximal words ‘*that\there*’ or the distal words ‘*this\here*’ are hard to acquire and comprehend. It has been predicted by Clark and Sengul’s (1977) that ‘*this*’ and ‘*here*’ are easier to acquire and comprehend than ‘*that*’ and ‘*there*’. This is because the first pair is proximal to a speaker, and the shift of reference is less than the second pair. The results of the second experiment showed that children did well with ‘*here\this*’ when they were sitting beside the experimenter. However, when they were sitting opposite each other, they did well with ‘*there*’ and ‘*that*’. On the contrary, Tfouni and Klatzky (1983) gave the reason why ‘*that*’ and ‘*there*’ might be easier than ‘*this*’ and ‘*here*’. This is due to the fact that they are used in many different applications other than ‘*this*’ and ‘*here*’. For example, they can be used as a relative pronoun, a subordinating conjunction, and they appear in existential constructions. In addition, they can be used in a deictic sense or in an anaphoric sense.

Krasnoshchekova (2012) focused in her study on how Russian-speaking children acquire the system of demonstratives. Her study aims to show the process of building up the demonstrative system of Russian-speaking children like adult. The study used longitudinal observations of children’s speech in addition to parental diaries, audio and video recordings. There were 14 monolingual children (7 boys and 7 girls) who were aged 1–4. Their speech was recorded by their parents once a month. The results showed that the first demonstrative pair which produced based on the data is the locative adverbs ‘*there\here*’ in the age of 1,3 and 1,4; for example, ‘Dania there Dania’ which means ‘Dania! There is Dania!’. To be more precise, the demonstrative ‘*here*’ appeared immediately after ‘*there*’. Later, children started to use the pronoun ‘*this*’. After that, they produced ‘*that*’ which is paired with ‘*this*’ at the ages of 2,5 and 2,6.

The study of Webb and Abrahamson (1976) tested the children’s comprehension of the deictic pair ‘*this\that*’ through comprehension and production tasks. The sample contains 60 children of ages 4 and 7. They were divided into two groups based on their age. Each group was made up of 15 children of both genders. In the comprehension task, children were instructed to choose one of two toys based on the speaker’s instructions. The children and the experimenter first sat next to each other, then opposite to each other. In this task, there were two expectations. The first prediction which was confirmed is that children were expected to learn the spatial deictic words, and subsequently learn to use them from the experimenter’s perspective. The second prediction is that children can perform well with the deictic word ‘*that*’. However, the latter prediction was not confirmed when the experimenter and the children were sitting next to each other. In the production task, the experimenter replaced the toys with two different candies. She covered them by using paper cups to not distract the children’s attention from choosing a specific one. Then she asked a child, “Which candy do you want?.” If the child did not either utter the word or point to it, the experimenter asked the child again, stating, “Point to the candy you want and say either ‘this one’ or ‘that one’..” The result showed that children who are four-years-old have a bias toward objects near them, while seven-year-old children preferred to choose objects which are far away from them. Furthermore, two out of 60 children violated the restriction that ‘*this*’ could only refer to a nearby object. In terms of egocentrism, three-year-old children did not have a consistent linguistic reference point, while the seven-year-old children relied on themselves or the speaker as a point of reference.

Zhao (2007) conducted a study which aimed to investigate the children’s comprehension and production of demonstrative pronouns ‘*this*’ and ‘*that*’ in Mandarin Chinese. In addition, the study also attempted to show how children acquire the proximal and distal demonstratives. Eight nursery children, ages 3–6, who are monolingual Mandarin Chinese speakers took part in the study. They were used to test their developmental stages and age differences in their use of demonstrative pronouns. The egocentrism hypothesis of Piaget and the marking hypothesis of Clark were both tested in the study. The researcher used two trials in her study (same and different perspectives). She put two identical cartoon boxes on a table; one with candies and the other with cookies. Children were asked to choose one of these boxes in each trial. The study’s findings backed up the former hypothesis. It has been discovered that children under the age of six were unable to alter the deictic center when they and the experimenter had a different perspective. For the latter hypothesis, the results showed that children performed better with the marked ‘*this*’ than with the unmarked ‘*that*’. This study showed some inconsistent results because of the use of candies and cookies which distracted the children’s attention to the experimenter’s instructions. This happened with group 2 where a child chose her favorite snack instead of the requirement set by the experimenter. On the comprehension and production tasks, the results revealed that children did better with the proximal word than with the distal one. Both results contradicted Clark’s hypothesis which stated that children acquire the deictic word ‘*that*’ before ‘*this*’.

Similarly, the study of De Villiers and De Villiers (1974) used a procedure to test the children’s comprehension and production of some of the contrastive deixes, such as ‘*this\that*’ and ‘*here\there*’. The sample was 39 children whose ages ranged from 2.5 to 4.5. In order to create a comprehension and a production task, the researchers divided a table into two parts with a cup on each side. Regarding the comprehension task, the experimenter put an M&M under one of the cups and then asked a child to take it; for example, “The M&M is over here/there.” In the production task, the experimenter was blindfolded. He asked another participant to hide the M&M inside one of the cups. Then, he asked the child to tell him where the M&M is. The first task is done when the experimenter faced the child while the second task is done in two different ways: they were opposite and next each other. The results of the study showed some of children’s proximity bias. Unlike most of the studies, this study showed that 3 or 4-year-old children have the ability to take a speaker as a point of reference for the demonstratives ‘*this*’, ‘*that*’, ‘*here*’ and ‘*there*’ in both tasks. This result is against the evidence which stated that preschool children are egocentric in their point of view.

## Research questions

**Q1.** Is there any age effect on children’s comprehension and production of acquiring English demonstratives and locative adverbs as a second language?

**Q2.** Do children take the speaker as a point of reference?

**Q3**. Do children first acquire the proximal deixis ‘this\here’ or the distal ones ‘that\there’?

**Q4**. Which deictic pair ‘this\that’ or ‘here\there’ do children acquire first?

## Materials and methods

### Subjects

The total number of children who were involved in the study is 30. They are bilingual Arabic children who acquire English as a second language. They were divided into three groups based on their ages who are from 4 to 6 years old. Each group contains 10 children of both genders and they were chosen randomly. The children are from different Arabic nationalities, such as Saudi, Syrian, Sudanese, Egyptian, etc. The ages of the participants were chosen based on previous studies. The deictic words appear firstly with children at the age of two and a half (Grant, 1915; Nice, 1915; Rodrigo, 2004 as cited in Zhao, 2007), and they acquire them like adults not until the age of six or seven (Clark and Sengul 1977; Küntay and Özyürek, 2006).

### Stimuli

The total number of testing sentences which were used with each child is 16; eight in each task and four in each trial. In the comprehension task, there were eight testing sentences with the use of *this\that and here\there*. For example, the experimenter says to a child: “*the toy is here*” or “*the toy is in this cup*.” On the other hand, the production task contained four phrases in each trial in order to force children to produce the words instead of using their figures to point or giving an eye-gaze. For instance, the experimenter says: “*can you tell me where the candy is? Is it here or there? \Is it in that cup or this cup*?.” The order of the testing sentences was randomized by the experimenter.

### Materials

Two identical black paper cups on both sides of a table are used. In the comprehension task, one of the cups contained a small toy and a sticker. On the other hand, one of the cups in the production task contained only a piece of candy. The stickers and the candies were used in order to motivate children to think before they answer. In addition, two different puppets (monkey and frog) are used in order to avoid children from being bored; one is used in the first task while the other is used in the second task. Moreover, there is a use of a recorder in both tasks to make sure about the children’s responses in the production and comprehension tasks.

### Tasks

**Figure 1:**
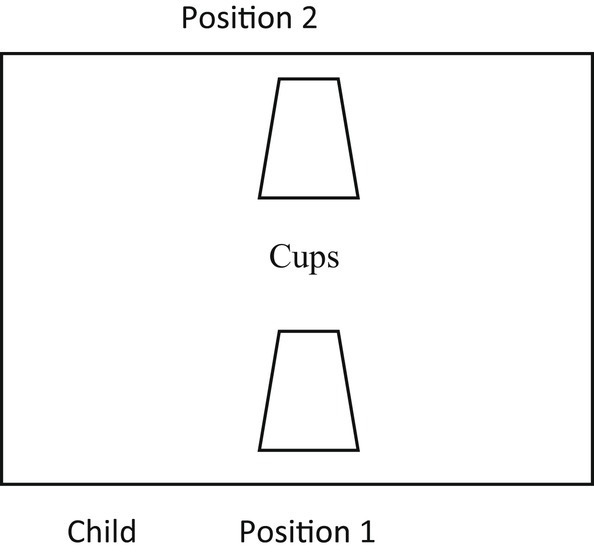
The situations used in both comprehension task and production task.

In this study, there are two types of tasks: comprehension task and production task. The first task tested the children’s comprehension of the words ‘*this*’, ‘*that*’, ‘*here*’, and ‘*there*’ in two different trials: same perspective and different perspective. On the other hand, the second task tested the children’s production of the same words in two different trials. These two tasks are based on the procedure of De Villiers and De Villiers (1974). In both tasks, there was a table which was divided into two parts to indicate far and near space. There were also two identical black paper cups which were put upside down, and one of them contained a toy and a sticker (in the comprehension task) and a candy (in the production task). The two cups were hand reachable in order to avoid any proximity bias. Unlike the original procedure of De Villiers and De Villiers (1974), the experimenter in this study used a puppet to utter testing sentences in order to avoid any non-linguistic cues, such as eye gaze. It is similar to the studies of Muşlu (2015) and González-Peña et al. (2020) where they used a puppet when they uttered the testing sentences to avoid any non-linguistic cues which may help children to choose the intended object (Figure 1).

### Procedure

Unlike the procedures of Webb and Abrahamson (1976), Clark and Sengul (1977) and Zhao (2007), the procedure of this study (hide-and-seek game) is very familiar with children which increased the natural communication between them. The experimenter tested each child individually in a small private room which contained a table and two chairs at the children’s school.

#### Comprehension task

At the beginning of the task, the experimenter played a recorder and told each child that they are going to play hide-and-seek game, and she\he had to listen to the puppet’s instructions in order to find the hidden toys. The task began with the experimenter sat next to the child (same perspective). Then, she asked the child to turn around and close her\his eyes while she was hiding a toy inside one of the cups. To avoid any auditory cues, the experimenter lifted both cups in every time. After that, the child opened her\his eyes and the experimenter used a puppet, either the frog or the monkey, to give the instructions. The use of a puppet helped the experimenter to avoid any non-linguistic cues by looking directly at the puppet, while the puppet is looking at the child, and its arms perform ambiguous gestures. In order to encourage the children to think and to avoid any proximity bias, the experimenter hid a sticker with a toy in one of the cups, and she gave the sticker to those children who answered correctly. If they do not, the puppet will take the stickers. In each trail, the experimenter uttered four testing sentences for each participant by using ‘*this*’, ‘*that*’, ‘*here*’, and ‘*there*’; for example, ‘the toy is in that cup’, or ‘the toy is here’. Thus, eight testing sentences are divided into two groups. Four testing sentences are uttered when the experimenter is sitting next to the child (same perspective), and the other four are uttered when the experimenter is sitting opposite to the child (different perspective).

#### Production task

At the beginning of this task, the experimenter informed each child that they are going to change the game slightly. The task began with the experimenter sitting next to the child (same perspective). First, the experimenter asked a volunteer assistant to help her conceal a piece of candy in one of the cups. The puppet and the experimenter covered their eyes while the child was watching the place of the candy. The assistant should hide candies in four specific places for each trial: twice inside the near cup and twice in the far cup. The instructions had been given to her before starting the task to avoid hiding candies in the same place and in the same trial three times in a row. Then, the experimenter and the puppet opened their eyes and asked the child about the place of the candy. The experimenter attempted to avoid non-linguistic responses from the children by asking them to choose between two deictic words; for example, the experimenter said “Is the candy here or there?” or “Is it in that cup or in this cup?.” So, the experimenter asked the children to make a choice between two deictic words in order to force them to use the linguistic cues and to avoid the non-linguistic ones. The aim of this task is to motivate children to produce the demonstrative words by asking them about the place\location of candies. If the children answer correctly, they can keep the candy. If they do not, the puppet will take the candy. Like the comprehension task, this task contains two trials: same perspectives and different perspectives.

## Data collection and analysis

The results of the study were collected through the observation of children’s responses and reactions in comprehension and production tasks in two trials (same perspective and different perspective). The data were illustrated in Tables 5, 6. Table 5 shows the results of children’s correct responses in the comprehension task in the two trials (same perspective and different perspective). As well as that, Table 6 represents the children’s responses in the production task in the two trials. The Tables show that children were divided into three different groups based on their age. Each group contained 10 children of the same age. Tables 5, 6 documented only the children’s correct responses.

**Table 5:** The number of children’s correct responses in the comprehension task.

	**Same Perspective**				**Different Perspective**			
	**This**	**That**	**Here**	**There**	**This**	**That**	**Here**	**There**
**4**	**7**	**1**	**6**	**7**	**9**	**5**	**6**	**5**
**5**	**5**	**6**	**9**	**8**	**7**	**9**	**9**	**8**
**6**	**7**	**5**	**8**	**8**	**8**	**9**	**8**	**8**

**Table 6:** The number of children’s correct responses in the production task.

	**Same Perspective**				**Different Perspective**			
	**This**	**That**	**Here**	**There**	**This**	**That**	**Here**	**There**
**4**	**8**	**6**	**7**	**4**	**6**	**4**	**7**	**3**
**5**	**6**	**6**	**8**	**7**	**2**	**2**	**4**	**5**
**6**	**8**	**8**	**10**	**9**	**1**	**5**	**5**	**5**

**TEST 3:** Same perspective (Table 5) and same perspective (Table 6). Group Statistics

	**Factor**			**N**			**Mean**	**Std. Deviation**	**Std. Error Mean**	
Data3	Same Perspective (Table 5)			12			6.4167	2.10878	0.60875	
	Same Perspective (Table 6)			12			7.2500	1.60255	0.46262	
Independent Samples Test										
		Levene’s Test for Equality of Variances		t-test for Equality of Means						
		F	Sig.	t	df	Sig. (2-tailed)	Mean Difference	Std. Error Difference	95% Confidence Interval of the Difference	
									Lower	Upper
Data3	Equal variances assumed	0.297	0.591	−1.090	22	0.288	−0.83333	0.76459	−2.41899	0.75233
	Equal variances not assumed			−1.090	20.528	0.288	−0.83333	0.76459	−2.42561	0.75895

**TEST 4:** Different perspective (Table 5) and different perspective (Table 6). Group Statistics

	**Factor**				**N**		**Mean**	**Std. Deviation**	**Std. Error Mean**	
Data4	Different Perspective (Table 5)				12		7.5833	1.50504	0.43447	
	Different Perspective (Table 6)				12		4.0833	1.78164	0.51432	
Independent Samples Test										
		Levene’s Test for Equality of Variances		t-test for Equality of Means						
		F	Sig.	t	df	Sig. (2-tailed)	Mean Difference	Std. Error Difference	95% Confidence Interval of the Difference	
									Lower	Upper
Data4	Equal variances assumed	0.280	0.602	5.199	22	0.000	3.50000	0.67326	2.10374	4.89626
	Equal variances not assumed			5.199	21.402	0.000	3.50000	0.67326	2.10147	4.89853

This study has adopted two different statistical methods (Two-Way ANOVA test and t-test) in order to analyze the data. In Two-Way ANOVA test, we tried to compare between the same perspective and the different perspective with different ages within the same task (comprehension and production task), but it did not provide any significant results. On the other hand, we applied t-test which provided interesting results.

## Results and discussion

The findings of the present study are presented in order to answer the research questions. The results are illustrated in Tests 1-4. Test 1 showed the result of the comprehension task in the same and different perspectives, while Test 2 showed the result of the production task in both same and different perspectives. Tables 3, 4 compared between the same perspectives or the different perspective in the two tasks (comprehension task and production task).

**TEST 1:** Same perspective (Table 5) and different perspective (Table 5). Group Statistics

	**Factor**				**N**	**Mean**	**Std. Deviation**	**Std. Error Mean**		
Data1	Same Perspective (Table 5)				12	6.4167	2.10878	0.60875		
	Different Perspective (Table 5)				12	7.5833	1.50504	0.43447		
Independent Samples Test										
		Levene’s Test for Equality of Variances		t-test for Equality of Means						
		F	Sig.	t	df	Sig. (2-tailed)	Mean Difference	Std. Error Difference	95% Confidence Interval of the Difference	
									Lower	Upper
Data1	Equal variances assumed	0.395	0.536	−1.560	22	0.133	−1.16667	0.74789	−2.71770	0.38437
	Equal variances not assumed			−1.560	19.898	0.135	−1.16667	0.74789	−2.72726	0.39393

**TEST 2:** Same perspective (Table 6) and different perspective (Table 6). Group Statistics

	**Factor**				**N**	**Mean**	**Std. Deviation**		**Std. Error Mean**	
Data2	Same Perspective (Table 6)				12	7.2500	1.60255		0.46262	
	Different Perspective (Table 6)				12	4.0833	1.78164		0.51432	
Independent Samples Test										
		Levene’s Test for Equality of Variances		t-test for Equality of Means						
		F	Sig.	t	df	Sig. (2-tailed)	Mean Difference	Std. Error Difference	95% Confidence Interval of the Difference	
									Lower	Upper
Data2	Equal variances assumed	0.180	0.675	4.578	22	0.000	3.16667	0.69176	1.73204	4.60129
	Equal variances not assumed			4.578	21.758	0.000	3.16667	0.69176	1.73111	4.60222

### Results of comprehension and production tasks in both trials

As seen in Table 5, children who are five and six-years-old have shown a little increased of correct responses in the comprehension task in both trials than those at age four. They actually scored 46 correct answers, while those at ages five and six scored 61. The results of four-year-old children can illustrate two different reasons. First, the results may go back to those children, especially boys, who tended to guess the place of the toy instead of thinking of what the puppets said. Second, they may not fully comprehend the meaning of the deictic words ‘*this*’, ‘*that*’ ‘*here*’ and ‘*there*’. In other words, the result of Test 1 did not provide any significant differences between the same perspective and the different perspective in the comprehension task.

On the other hand, Table 6 shows that four-year-old children got five more correct responses than those at age five who got 40 in both perspectives. The result of this task with age four shows irregular development. Most of them tended to guess the place of the candies or they chose the last word which the puppet said. According to the observation in the different perspective trial, a few numbers of 4 and 5-years-old children started to notice the difference between the deictic center. They noticed that when they answered incorrectly, the puppets took either the sticker or the candy. Then, they knew that the answer they chose was incorrect. The six-year-old children scored 51 correct responses. The result of Test 2 showed that there is a difference between the same perspective and the different perspective in the production task. Children actually in the production task showed some understanding of the spatial deixes in the same and different perspectives.

The results of Tests 1, 2 illustrate that children of all ages have a good ability to comprehend the spatial deixis ‘*this*’, ‘*that*’, ‘*here*’ and ‘*there*’ than to use (produce) them. In other words, the total results of each age group in each task reveal that the comprehension task has a higher number of correct responses than the production task. According to the children’s responses in both tasks, they acquired first the proximal words ‘*this*’ and ‘*here*’ then the distal words ‘*that*’ and ‘*there*’. According to Clark and Sengul (1977), the proximal words are easier to acquire than the distal ones. Specifically, children scored more correct answers with the word ‘*here*’ than ‘*there*’, and ‘*this*’ than ‘*that*’. In addition, all children acquired the deictic contrast ‘*here\there*’ before ‘*this\that*’. As Clark and Sengul (1977) and Zhao (2007) found, children acquired first ‘*here\there*’ then ‘*this\that*’. However, the results of Krasnoshchekova (2012) showed that children at earlier ages acquired the distal word ‘*there*’ than the proximal one ‘*here*’, then they acquired ‘*this*’ before ‘*that*’. Similarly, the study results of Küntay and Özyürek (2006) showed that children do not acquire ‘*this*’ and ‘*that*’ until the age of six. Through the observation, many children at ages four and five did not notice the difference between proximal and distal deixes, especially in the production task when they use their fingers to point. They used ‘*here*’ and ‘*this*’ to indicate both far and near objects\places. When the experimenter in the production task told them not to use their hands or give an eye-gaze, they started noticing the difference between far and near objects and they gave a correct answer. In the current study, children responded to most of the spatial deixes correctly in the same perspective trials, while they responded incorrectly in the different perspective trial. In addition, when they were next and opposite the experimenter, they did well with ‘*here*’. On the other hand, their responses with other words were varied.

Unlike the studies of De Villiers and De Villiers (1974), Webb and Abrahamson (1976) and Clark and Sengul (1977), there was no proximity bias happened with all ages in the present study. Although Clark and Sengul (1977) put the cups near the children in order to avoid any proximity bias, they still chose the nearest cup to them. However, even the cups in present study were hand-reachable to the children, there was no bias toward near objects. So, there was no occurrence of proximity bias with all ages. If children did not understand what the puppets said, they tended to guess which cup contained the toy and the candy. Guessing was more widespread among the younger children than the older ones. In both tasks, a small number of four-year-old children did not listen to the puppets’ instructions. They quickly chose a cup when they opened their eyes. Some children chose the last word which the puppet said in the production task when they felt that they were unable to choose the correct word.

Unlike the studies of Tfouni and Klatzky (1983) and Muşlu (2015), the present study does not use eye-gaze and pointing to help children in the tasks. In fact, the results of their studies showed that the use of non-linguistic expressions can affect the children’s ability to perform correctly. Similarly, Zhao (2007) mentioned that the non-linguistic expressions, such as pointing and eye-gaze, play a main role in helping children to understand the meaning of deictic words. Like the study of Gonzalez Pena (2020), the present study dealt with this issue by giving only the instructions without the use of non-linguistic expressions in order to have a clear picture of the children’s development stages of acquiring English spatial deixis. It is also not allowed for children to use non-linguistic expressions. When children used their hands to point to one of the cups, we told them not to use their hands to point and they had to choose between the phrases which the puppet provided.

### Result of the same perspective and the different perspective

Tests 3, 4 focused on the children’s understanding of the same and different perspectives. Test 3 showed the result of the same perspective in both Tables 5, 6, and the result did not show any significant differences. To be more specific, six-year-old children clearly have more control of spatial deixis in both perspectives than four-and five-year-old children. When comparing the responses of children in the same perspectives, it has been noticed that four-year-old children performed better than those at age five. After that, the performance increased again at age six which shows the U-shaped pattern of development. The result is surprising that four-year-old children did better than those at age five. Similarly, this happened in the study of Muşlu (2015) in which children at age four did better than those at age three and five, and she explained this issue in her paper by saying that “[T]he learner first learns a given target behavior, then unlearns it and finally learns it” (p: 423). Moreover, Long (1990) also explained the U-shaped pattern of development by stating, “Progress is not linear; backsliding is common, giving rise to so-called U-shaped behavior observed in first and second language acquisition” (p: 659).

On the other hand, Test 4, which provided the result of the different perspective in both tables, showed a significant result. Children showed an increase in understanding the changes of the deictic center with age. In the present study, when the experimenter changed her place and sat opposite to the children, they started to notice that there was a problem. They get confused about which perspective to take. Clark and Sengul (1977) have mentioned that children face some problems in changing the deictic center. The result is not surprising because it is consistent with the results of the previous studies (Clark and Sengul, 1977; Zhao, 2007; Muşlu, 2015).

In addition, Zhao (2007) concluded her results by mentioning that children think in an egocentric way which may be considered as a universal phenomenon that obstructs children’s acquisition of deictic words. In other words, children who are under the age of six do not have the ability to change the deictic center when they and the speaker have a different perspective (Zhao, 2007). Just as Zhao (2007), Meltem Muşlu (2015) mentioned in her study that children between the ages of 2 and 7 think in an egocentric way. In other words, children did not acknowledge any other perspectives except their own. Thus, they failed to recognize the speaker-center in the different perspective. However, the observation of the two tasks in the present study showed that most of the children noticed that there was a problem in their comprehension and production of the spatial deixis when the experimenter changed her place and sat opposite to them. They noticed that there is something wrong happened when the experimenter changed her place. For example, when the puppets got the candies and stickers instead of giving them to the children, they noticed that their answers were incorrect. Similar to the results of the current study, De Villiers and De Villiers (1974) stated that children showed a simple ability to take a speaker as a point of reference in the second trial (different perspective). Thus, children are not totally egocentric.

## Conclusion

To sum up, all languages, especially deixis words, are designed for face-to-face communication in daily life which cannot be separated from the context of utterance. The current study aims to investigate how Arabic children between the ages of four, five and six acquire the English spatial deixis ‘*this*’, ‘*that*’, ‘*here*’ and ‘*there*’. In order to investigate that, a hide-and-seek game was used to observe and record the children’s responses about English spatial deixis. The results indicate that children, unlike the previous studies, did not show any proximity bias regarding their choice of the cups. Instead, they tended to guess the place of candies and stickers when they did not understand what the puppets said. Moreover, there was a U-shaped pattern of development in the same perspective in both tasks, while they showed an increase with age in different perspective. The results also showed that children first acquire the proximal words ‘*here\this*’ than the distal ones ‘*there\that*’. In addition, they also acquire the deictic pair ‘*here\there*’ before ‘*this\that*’. The results of the study can show some inconsistent results because children, especially those at age four, tended to guess the place of the candy and sticker instead of thinking.

### Limitations

The present paper attempts to provide a clear picture of the acquisition process of spatial deixis with children at ages four, five and six. However, the study’s results cannot be generalized because of some limitations regarding the samples. Further studies should include a larger number of children from different ages.

## Data availability statement

The original contributions presented in the study are included in the article/supplementary material, further inquiries can be directed to the corresponding author.

## Author contributions

HiA and HaA developed this paper. HaA was responsible for the literature review and setting up the theoretical framework and methodological part. HiA was in charge of collecting and analyzing the data, displaying the findings, and writing the discussion section. All authors contributed to the article and approved the submitted version.

## Conflict of interest

The authors declare that the research was conducted in the absence of any commercial or financial relationships that could be construed as a potential conflict of interest.

## Publisher’s note

All claims expressed in this article are solely those of the authors and do not necessarily represent those of their affiliated organizations, or those of the publisher, the editors and the reviewers. Any product that may be evaluated in this article, or claim that may be made by its manufacturer, is not guaranteed or endorsed by the publisher.
